# Research on the construction of growth models for dominant tree species in the Manas River Basin, Xinjiang

**DOI:** 10.7717/peerj.20650

**Published:** 2026-02-16

**Authors:** ZhongQiong Zhao, Mei Zan, Jian Ke, Jia Zhou, Lili Zhai, Cong Xue, Shunfa Yang, Yu Dong, Yuntong Liu

**Affiliations:** 1 Xinjiang Normal University, Urumqi, Xinjiang Uygur Autonomous Region, China; 2 Natural Science Foundation of Xinjiang Uygur Autonomous Region, Urumqi, Xinjiang Uygur Autonomous Region, China; 3 National Natural Science Foundation of China, Urumqi, Xinjiang Uygur Autonomous Region, China

**Keywords:** Diameter at breast height (DBH), Tree height, Forest growth model, Manas River Basin, Feature variable, Stand age

## Abstract

Research on forest growth models is not only crucial for regional ecological security and the optimal allocation of water and carbon resources but is also a key component in the study of carbon cycling in arid regions, holding scientific and practical significance for addressing climate change and promoting green sustainable development. Therefore, this study takes the Manas River Basin in Xinjiang as an example and based on the 2011 forest resource survey data from the Manas River Basin, constructs basic growth models for the diameter at breast height (DBH)-height and age-DBH relationships for five dominant tree types: Spruce, Poplar, Mixed wood, Sand jujube, and *Populus euphratica*. The optimal basic models for each types are selected. Secondly, climate factors (annual precipitation, Minimum of Daily Maximum Temperature, TXn) and topographic factors (Digital Elevation Model; DEM) are introduced into the optimal models to construct multivariate nonlinear forest growth models. Finally, deep learning is used to optimize the overall accuracy of the models. The results show that the optimal DBH-height models for Spruce, Poplar, Sand jujube, and *Populus euphratica* are S-curve models, while the optimal DBH-height model for Mixed wood is a logarithmic model. The optimal age-DBH models for Poplar and *Populus euphratica* are S-curve models, whereas the optimal age-DBH basic models for Spruce, Mixed wood, and Sand jujube are growth model, linear model, and logistics model, respectively. The overall accuracy of the multivariate nonlinear forest growth models is improved, with the highest R^2^ reaching 0.890 and the average RMSE increasing by 10.590, mainly due to the decrease in model accuracy for some tree types caused by random factors. Lastly, compared to the basic models and multivariate nonlinear forest growth models, the deep learning approach demonstrates the best performance, with the highest correlation coefficient reaching 0.960. Overall, by constructing forest growth models for five main dominant tree types in the Manas River Basin in Xinjiang, the optimal forest management strategies in the region can be determined, which helps to formulate targeted forest management strategies, effectively address the allocation of carbon and water resources, and promote healthy and sustainable forest development.

## Introduction

Forests are an important part of terrestrial ecosystems, playing a crucial role in ecological functions, energy flow, climate regulation, and material and energy resources, among many other aspects (Wingfield, 2015). Plantations comprised of a single tree types exhibit simplified ecosystem structures and lower biodiversity, often resulting in reduced stability and resilience, especially given the current occurrence of extreme weather conditions such as high temperatures and drought, making the research on natural forests, particularly natural Mixed wood, even more urgent (Bremer & Farley, 2010). Therefore, understanding the dynamic changes of forest resources has become a current hot topic and challenge. Tree height, diameter at breast height (DBH), and forest age, as important factors describing stand growth (Deng, 2023), play a decisive role in tree productivity and vitality, reflecting the long-term competition levels during tree growth. They are essential factors that need to be grasped (Yang, Lin & Sun, 2018). By establishing a basic forest model, we can simulate stand growth patterns and estimate stand growth and harvest (Tu, 2020). Measuring diameter at breast height (DBH) is relatively easier to achieve compared to measuring tree height. By establishing regression models between DBH and tree height, we can estimate the difficult-to-measure tree height using the easily measured DBH. Meanwhile, forest ecosystems are sensitive to changes in climate, precipitation, light, and future climate changes will inevitably affect tree growth (Zang, 2016; Sang, 2019), therefore, in studies conducted in different regions, tree growth is influenced by various factors such as climate, leading to different fitting results of forest growth models. The research on basic models is fundamental for understanding the growth status of forests. By establishing Logistic models and Richard’s curve regression models, we can fit the growth models of stand age (a) and stand volume per hectare (M) for small forest (Zhang, Wang & Lu, 2019) stands (Liu, 2011). Calculate the annual increment, and average increment of stand in the forest area. Taking into account the influence of other variables such as climate on trees, a stand competition index variable is introduced into the model (Huang, 2022). Compare the effects of individual tree variables, branch variables, and stand competition variables on branch growth, and construct a nonlinear mixed-effects model at the individual tree level (Liu et al., 2024). In the context of global warming, studying the impact of climate change on tree growth and the response to climate change holds significant theoretical importance (Ning, 2023). Deep learning models are still relatively rare but becoming a trending approach in the field of forest growth research. Three different machine learning models, namely Artificial Neural Networks (ANN), Support Vector Machines (SVM), and Random Forest (RF) models were used to predict tree growth at the plot level in Brazilian Atlantic Forests (Rocha, 2024). It indicated that the Random Forest method significantly outperformed the other methods in modeling forest growth. In the study, Metsaranta (2024) pointed out that some models used in forest growth research may have limited effectiveness if they do not fully consider climate and other environmental variables. Therefore, there is an urgent need for climate-sensitive growth and yield models (CSGYMs) to support forest management decisions. Deep learning still requires in-depth exploration and research in the field of forest growth modeling. Previous research on basic forest models has reached a relatively mature stage, revealing significant differences in the function models applicable to different regions and tree types. Furthermore, given that tree growth is easily influenced by external environmental factors such as topography and climate, studies on tree growth have primarily focused on analyzing tree mortality, self-thinning, distribution, and productivity (Zhang, Wang & Lu, 2019; Lu, Zhang & Lei, 2015). However, there has been limited research on the refined study of the growth of dominant tree types within specific small areas, which hinders the accurate capture and understanding of tree growth trends. Therefore, this article focuses on the research of forest growth models in the Manas River Basin of Xinjiang, aiming to achieve the protection of dominant tree types in the region and to take corresponding measures in response to environmental changes.

In recent years, due to rapid population growth and accelerated urbanization, issues such as irrational water resource utilization, sharp declines in biodiversity, land desertification, and degradation of forest resources have become increasingly prominent (Jiao et al., 2022). Based on the forest resource survey data from the Manas River Basin in Xinjiang, this article establishes various basic models for dominant tree types. The forest resource survey data from 2011 is selected for constructing the basic forest models, which contains detailed forest information such as forest type, tree height, forest age, and sample plot area. This data has the advantages of high accuracy and strong timeliness. Meanwhile, considering the impact of natural factors on tree growth, topographic and climatic factors are introduced into the optimal basic mode to establish a multivariate nonlinear forest growth model. Finally, this paper uses deep learning methods to calculate the correlation coefficients of the models. The aim is to explore the influence of natural factors on tree growth and to provide management and restoration strategies for forest growth and production operations in the Manas River Basin in Xinjiang. In summary, this paper takes the Manas River Basin as the study area and obtains the optimal basic model for dominant tree types. By combining climatic and topographic factors, a multivariate nonlinear forest growth model is obtained, and the results are compared with those from deep learning. The findings of this study provide a data basis and reference for the construction and development of forest tree models in the Manas River Basin in Xinjiang.

## Materials and Methods

### Overview of the study area

**Figure 1:**
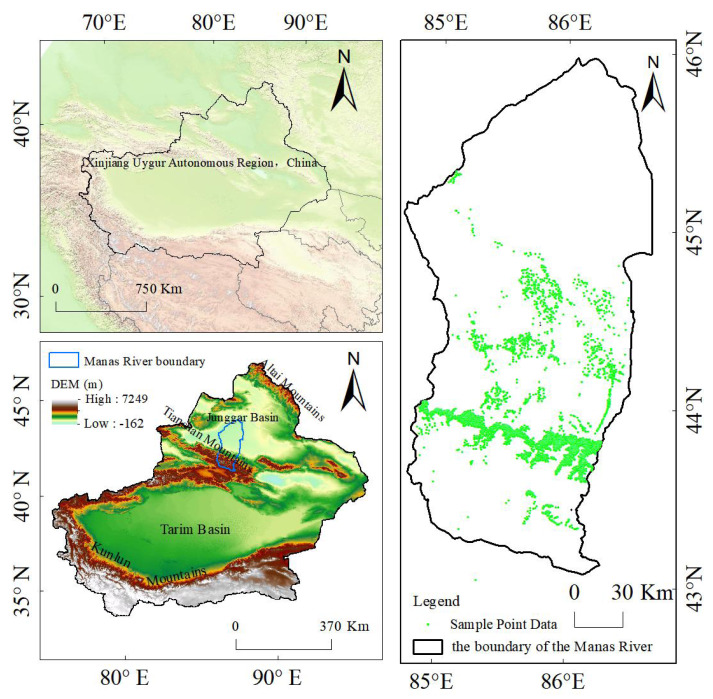
Overview of the geographical location of the study area and sample data points.

The Manas River basin in Xinjiang is located in the southwest of the Junggar Basin and the middle section of the northern foot of the Tianshan Mountains, with geographical coordinates ranging from 43°27 2 to 45°21 2 N and 85°01 2 to 86°32 2 E, as shown in Fig. 1. The Manas River Basin in Xinjiang, as the largest oasis farming region in Xinjiang and the fourth largest irrigated agricultural area in China (Kang et al., 2024), is one of the important sources of water for daily use, agricultural irrigation, and industrial production (Ma, 2024a; Ma, 2024b). The main rivers within the basin include the Manas River, Jingou River, Taxi River, Bayingou River, and others. Among them, the Manas River, with a total length of approximately 450 km, originates from the northern foot of the Tianshan Mountains. The vegetation coverage within the basin is relatively good, and the sediment content of the river is relatively low (Ma, 2024a; Ma, 2024b). The altitude of the Manas River basin gradually decreases from south to north, encompassing various landform types such as glacial land forms, alluvial-proluvial land forms in the folded hilly areas, meandering land forms in the plain areas, and terminal lake land forms. The basin features a typical mountain-oasis-desert landscape structure with prominent vertical zonality, and the main type of land use is grassland. The plain area of the basin has a temperate continental arid climate with large temperature differences, an annual average temperature of 6.8 °C, an annual average precipitation of 110–200 mm, and an annual average potential evaporation of 1,500–2,100 mm (Yin, Xu & Hu, 2024). This basin is a major agricultural area in Xinjiang, with cotton, tomatoes, and grapes being important crops in the basin (Zhao & Wang, 2024). The main dominant tree types in the Manas River basin include Spruce (*Picea asperata Mast.*), Elm (*Ulmus pumila L.*), Poplar (*Populus L.*), Euphrates poplar (*Populus euphratica*), Sand jujube (*Elaeagnus angustifolia Linn.*), Black locust (*Robinia pseudoacacia L.*), Ash (*Fraxinus chinensis Roxb.*) and Willow (*Salix*). Among them, Spruce is the most abundant, due to insufficient individual numbers in the study area, reliable species-specific models could not be established for Elm *(Ulmus spp.)*, Black locust (*Robinia pseudoacacia)*, Ash (*Fraxinus spp.)*, and Willow (*Salix spp.*). To ensure analytical reliability, we consolidated these tree species into a composite category termed ‘Mixed wood’. The rationale for this merger is based on their similar ecological niche and functional attributes in the habitat of the region. First of all, these species are all common non-zonal deciduous broad-leaved tree species in the desert-oasis transition zone of the southern margin of Junggar Basin in northern Xinjiang (Liu, 2023). Secondly, there are similarities in functional traits and ecological strategies. In the Manas River basin and similar arid zones of Central Asia, they are mostly mesophytic or mildly drought-tolerant aquatic wetland tree species, and their distribution is closely related to the depth of groundwater and river valley zone (Bai, Hu & Bu, 2023). Finally, as early or mid-successional tree species, they exhibit similar ecological response strategies to water resource changes (such as river diversion and groundwater extraction) and land use disturbances (Xie, He & Xie, 2024).

### Data sources and preprocessing

#### Forest resource inventory data

Forest resource survey data are obtained by conducting repeated surveys of the same area of forest resources on a regular basis, in order to grasp the quantity, quality, and growth changes of the forest resources. They serve as an important basis for understanding the current status and dynamic changes of forest resources and are essential data for establishing forest growth models (Chen et al., 2023). The data used in this study include the forest resource survey data of Xinjiang (abbreviated as “Class I Inventory”, with a survey cycle of 5 years). In 2011, Xinjiang conducted the 8th Class I Inventory, employing a systematic sampling method to lay out tree measurement plots on topographic maps at intervals of 4 km × 6 km in mountainous and 2 km × 3 km in plain areas. Within these plots, fixed sample plots were established as squares measuring 28.28  ×  28.28 m, covering an area of 0.07 ha. The “Class I Inventory” field data meticulously documented information such as forest type, tree height, stand age, and plot area, possessing advantages of high accuracy and strong timeliness (Du, Chen & Li, 2023). In this paper, the data were randomly divided into modeling samples and validation samples, accounting for 70% and 30% of the total samples, respectively. Statistical analysis of the sample data was conducted using relative professional software, with detailed statistical results presented in Tables 1 and 2. Tables 1 and 2 show the descriptive statistics of key variables in the modeling sample and the testing sample respectively.

**Table 1:** Statistical results of the modeling sample data.

Tree species	Diameter at Breast Height/cm				Tree height/cm				Stand age/s			
	Mean value	Standard Error (SE)	Minimum value	Maximum value	Mean value	SE	Minimum value	Maximum value	Mean value	SE	Minimum value	Maximum value
Spruce	20.47	6.58	6	40	15.51	4.27	3	9.90	112.91	34.69	16	187
Mixed wood	13.97	7.86	3	40	7.30	2.82	3	15.00	26.29	11.87	11	55
Poplar	13.48	4.77	6	40	11.67	3.82	4	22.00	20.58	6.27	9	72
Sand jujube	10.10	4.56	6	18	5.10	2.48	2	11.00	16.37	9.85	11	25
*Populus euphratica*	13.82	3.66	6	26	7.95	2.52	3	12.00	29.18	3.76	13	50

**Table 2:** Statistical results of the test sample data.

Tree species	Diameter at Breast Height/cm				Tree height/cm				Stand age/s			
	Mean value	Standard Error (SE)	Minimum value	Maximum value	Mean value	SE	Minimum value	Maximum value	Mean value	SE	Minimum value	Maximum value
Spruce	20.38	6.56	6	38	15.55	4.26	4	26.30	111.52	34.51	22	187
Mixed wood	12.95	7.83	3	30	6.68	2.77	3	15.00	26.88	12.29	9	57
Poplar	13.39	4.62	6	36	12.24	3.69	4	20.00	21.47	5.84	9	72
Sand jujube	8.50	4.12	6	14	6.63	2.47	5	12.00	15.25	10.81	13	20
*Populus euphratica*	15.78	3.70	12	18	9.22	2.75	7	12.00	29.78	4.07	11	39

#### Other related data

The vector boundary data for the study area used in this paper were obtained from the Resource and Environment Science and Data Platform (https://www.resdc.cn/Default.aspx). ArcGIS was employed to perform tasks such as clipping and projection transformation for the study area. The Digital Elevation Model (DEM) data originated from the Geospatial Data Cloud Platform (https://www.gscloud.cn/). With an annual temporal resolution and a spatial resolution of one km, this data exhibits strong reality and other advantages (Wang, 2022). The climate factor data used in this study were sourced from the Global Change Science Data Publishing & Repository (https://www.geodoi.ac.cn/WebCn/doi.aspx?Id=3582), which provides daily temperature data from all stations in China. The daily temperature grid data nationwide were interpolated using the inverse distance weighting (IDW) method. Annual temperature data were then obtained by averaging the daily temperature grid data within the administrative boundaries of prefecture-level cities, with a spatial resolution of 1km for all scales. For the raster datasets of annual precipitation (P), vapor pressure deficit (VPD), and minimum of daily maximum temperature (TXn) for the year 2011, obtained through the ArcGIS software, coordinate system transformations were conducted to ensure that the input raster layers and point layers shared the same coordinate system. Subsequently, the point value extraction method in spatial analysis, using the center value of the sampled pixel as the default option, was employed to extract corresponding climate and topographic data for the forest modeling sample points. The extracted data were then statistically analyzed and organized using Excel, ultimately generating corresponding vector data results for various climate and topographic factors. These data were primarily used for parameter selection and model construction in the multiple nonlinear forest growth model.

### Research methods

#### Establishment of basic models

In this study, SPSS 27.0 (IBM Corp., Armonk, NY, USA) and Matlab R2023a (The MathWorks, Natick, MA, USA) software were used to establish basic forest growth models such as diameter at breast height (DBH)—tree height and stand age—DBH based on sample plot observation data, with specific forms shown in Table 3. During the construction of the classic Logistic model describing forest growth, the nlinfit function was employed to optimize the relevant parameters (Wang, 2019), resulting in optimal theoretical models for the relationships between DBH and tree height, as well as stand age and DBH for each tree types. The model used can be found in Table 3.

**Table 3:** Model formula.

Model name	Model formula
Linear model	*y* = *b*_0_ + *b*_1_x
Logarithmic curve model	*y* = *b*_0_ + *b*_1_ln*x*
Reciprocal model	$y=b0+ \frac{{b}_{1}}{x} $
Quadratic polynomial model	Y = *b*_0_ + *b*_1_*x* + *b*_2_*x*^2^
Cubic polynomial model	*y* = *b*_0_ + *b*_1_*x* + *b*_2_*x*^2^ + *b*_3_*x*^3^
S-curve model	*y* = e^(*b*0+*b*1/*x*)^
Growth model	*y* = *e*^*b*_0_+*b*_1_*x*^
Logistic model	$~y= \frac{{b}_{1}}{1+\exp [-{b}_{2}\mathrm{\ast }(x-{b}_{3})]} ~$(where *b*_1_, *b*_2_, and *b*_3_ are the relevant parameters)

#### Establishment of multivariate nonlinear forest growth models

Apart from being influenced by the intrinsic characteristics of tree types, factors such as competition, site quality, and climate also have significant impacts on tree growth. To further improve the accuracy of the forest growth model in the study area, this article first conducted correlation analysis between annual precipitation, elevation, maximum temperature, minimum temperature, and other factors with diameter at breast height (DBH). Variables strongly correlated with DBH were selected to enter the optimal basic model. Then, a multiple nonlinear forest growth model was constructed (Wu, 2022). The specific form of the multiple nonlinear forest growth model is shown in Table 4. This provides theoretical basis and data support for tree growth research and management in the region.

To further improve the prediction accuracy of the model, this article conducts point-to-point extraction of raster data and forest resource modeling data. In order to quantify the strength of the association between these factors and the diameter at breast height (DBH), we perform statistical correlation analysis between the extracted geoclimatic factors, such as annual precipitation (P), vapor pressure deficit (VPD), maximum temperature extreme (TXx), minimum temperature extreme (TXn), maximum of minimum daily temperature (TNx), minimum of minimum daily temperature (TNn), as well as topographic factors like DEM data, and the DBH. Statistical analysis of the relationship between site factors and climate factors. Using correlation analysis techniques in statistics and we calculate the correlation coefficients between each of the aforementioned geoclimatic and topographic factors and the tree DBH one by one. This step aims to assess the linear association strength between each factor and DBH size. The various factors are summarized in Table 5. Through comparative analysis of the correlation coefficients between the geoclimatic and topographic factors and DBH, we select the three variables with the strongest correlation with DBH and include them as core independent variables in the basic model. Based on this, we further develop a multivariate nonlinear forest growth model, which can more comprehensively reflect the complex influence of geoclimatic and topographic conditions on forest growth dynamics.

**Table 4:** Form of multivariate nonlinear forest growth model.

Model name	Model formula
Multivariate linear model	*y* = *b*_0_ + *b*_1_*x*_1_ + *b*_2_*x*_2_ + …*b*_*n*_*x*_*n*_
Multivariate logarithmic curve model	*y* = *b*_0_ + *b*_1_ln*x*_1_ + *b*_3_*x*_2_ + …*b*_*n*_*x*_*n*_)
Multivariate reciprocal model	$y=b0+ \frac{{b}_{1}}{{x}_{1}} +{b}_{2}{x}_{2}+\ldots +{b}_{n}{x}_{n}$
Multivariate quadratic polynomial model	$\mathrm{Y}={b}_{0}+{b}_{1}{x}_{1}+{b}_{2}{x}_{1}^{2}+{b}_{3}{x}_{2}+{b}_{4}{x}_{3}+\ldots +{b}_{n}{x}_{n-1}$
Multivariate cubic polynomial model	$y={b}_{0}+{b}_{1}{x}_{1}+{b}_{2}{x}_{1}^{2}+{b}_{3}{x}_{1}^{3}+{b}_{4}{x}_{2}+{b}_{5}{x}_{3}+{b}_{6}{x}_{4}+\ldots +{b}_{n}{x}_{n-2}$
Multivariate S-curve model	*y* = e^(*b*0+*b*1/*x*_1_+*b*_2_*x*_2_+*b*_3_*x*_3_+…+*b*_*n*_*x*_*n*_)^
Multivariate growth model	*y* = *e*^*b*_0_+*b*_1_*x*_1_+*b*_2_*x*_2_+*b*_3_*x*_3_+…+*b*_*n*_*x*_*n*_^
Multivariate Logistic model	$y= \frac{{b}_{1}}{1+\exp (-{b}_{2}\mathrm{\ast }({x}_{1}-{b}_{3}))+{b}_{4}{x}_{2}+{b}_{5}{x}_{3}+\ldots +{b}_{n}{x}_{n-2}} $

**Table 5:** Estimation results of parameters for the basic diameter-at-breast-height-tree height model.

Variable	Environmental covariates	Maximum	Minimum	Average
Diameter at breast height/cm	DBH	40.000	3.000	19.040
Tree height/m	H	27.000	2.000	14.680
Net Primary Productivity/g C/m eyr	NPP	74.190	4.293	10.770
Biomass/g/^3^	V	27,713.480	0.723	2,144.300
Stock volume/m^3^	M	38,830.000	1.000	3,072.040
Elevation/m	DEM	3,587.000	220.000	1,877.290
Annual precipitation/mm	P	438.700	143.200	291.510
Vapor pressure deficit/kPa	VPD	125.670	−9999.000	45.880
Maximum temperature extreme/°C	TXx	41.120	30.350	38.020
Lowest maximum temperature/°C	TXn	−19.760	−27.560	24.230
Highest minimum temperature/°C	TNx	26.110	17.870	24.790
Lowest minimum temperature/°C	TNn	−32.530	−37.500	−33.780
Mean temperature difference/°C	DTR	12.050	9.990	10.700
Peak daily temperatures in summer/°C	SU	146.000	67.000	126.450
Daily highs during icy days/°C	ID	112.000	95.000	102.940
Duration of warm days/day	WSDI	13.000	4.000	7.450
Duration of continuous cold days/day	CSDI	19.000	9.000	14.570

#### Deep learning model

Machine learning methods are a class of intelligent data analysis techniques based on big data. Compared to traditional modeling methods, machine learning methods can achieve more accurate simulations and predictions. Deep learning methods, as an important branch of machine learning, are advanced machine learning techniques based on neural networks. They mimic the working manner of human brain neural networks, performing high-level abstractions of data through multiple layers of nonlinear transformations to discover complex patterns within the data (Niu, 2009). This paper employs deep learning methods to calculate the correlation coefficients for the basic models of diameter at breast height (DBH)—tree height and age—DBH for dominant tree types in the Manas River Basin of Xinjiang. We adopted a feedforward neural network with a single hidden layer containing 10 neurons, using the hyperbolic tangent function as the activation function. The output layer employed a linear activation function. The model was trained using the Levenberg–Marquardt algorithm with a learning rate of 0.01. In order to effectively prevent overfitting, we introduce L2 regularization (weight decay coefficient is 0.001), and monitor early stopping through the verification set during the training process to retain the model with the best generalization performance. To ensure the independence and reliability of the evaluation, we strictly divided the dataset into a training set (70%), a validation set (15%), and a test set (15%). All reported accuracy metrics are based on results from the test set, thereby avoiding overfitting-induced performance overestimation. Based on the introduction of variables such as climatic factors, the same method is used to calculate the correlation coefficients of the models. Compared to basic models and multiple nonlinear forest growth models, deep learning methods exhibit higher accuracy in establishing forest growth models. By utilizing a fully connected neural network constructed within the deep learning framework, combined with environmental factors, highly accurate predictions of DBH can be obtained. Many studies have demonstrated the feasibility of deep learning models in predicting tree growth (Zhang, Gu & Dong, 2014).

#### Model validation

The optimal models for DBH-tree height and age-DBH for each dominant tree types are obtained through the coefficient of determination R^2^. Then, the measured validation sample data for each main tree types are substituted into the obtained optimal DBH—tree height and age—DBH models. Through validation and calculation, the predicted values of tree height (DBH) for each tree types are obtained. By comparing the measured values of tree height (DBH) in the validation sample data, and calculating validation accuracy indicators such as the coefficient of determination and root mean square error for model validation, the evaluation criteria are established. The calculation formulas are as follows (Lu et al., 2015):

Relative:

(1) \begin{eqnarray*}\begin{array}{@{}c@{}} \displaystyle {\mathrm{R}}^{2}=1- \frac{\sum _{i=1}^{n}{ \left( {y}_{i}-{\hat {y}}_{i} \right) }^{2}}{\sum _{i=1}^{n}{ \left( {y}_{i}-{\bar {y}}_{i} \right) }^{2}} . \end{array}\end{eqnarray*}

Root Mean Squared Error:

(2) \begin{eqnarray*}\begin{array}{@{}c@{}} \displaystyle RMSE=\sqrt{ \frac{\sum _{i=1}^{n}{ \left( \hat {y}i-yi \right) }^{2}}{n-p} }. \end{array}\end{eqnarray*}

Mean Relative Error:

(3) \begin{eqnarray*}\begin{array}{@{}c@{}} \displaystyle MRE= \frac{\sum _{i=1}^{n} \frac{\hat {{y}_{i}}-{y}_{i}}{{y}_{i}} }{n} \end{array}.\end{eqnarray*}

Mean Error:

(4) \begin{eqnarray*}\begin{array}{@{}c@{}} \displaystyle \mathrm{ME}= \frac{\sum _{\mathrm{i}=1}^{\mathrm{n}}\hat {{y}_{i}}-{y}_{i}}{\mathrm{n}} \end{array}.\end{eqnarray*}

In the formula: $\hat {y}$ represents the predicted value of tree height (or DBH). y_i_ represents the measured value of tree height (or DBH). $\bar {y}$ presents the average value of tree height (or DBH). *n* represents the number of trees in the validation sample. And *p* represents the number of independent variables in the model.

## Result analysis

### Parameter testing and results of the basic model

#### Simulation results of the basic model for diameter at breast height tree height

**Figure 2:**
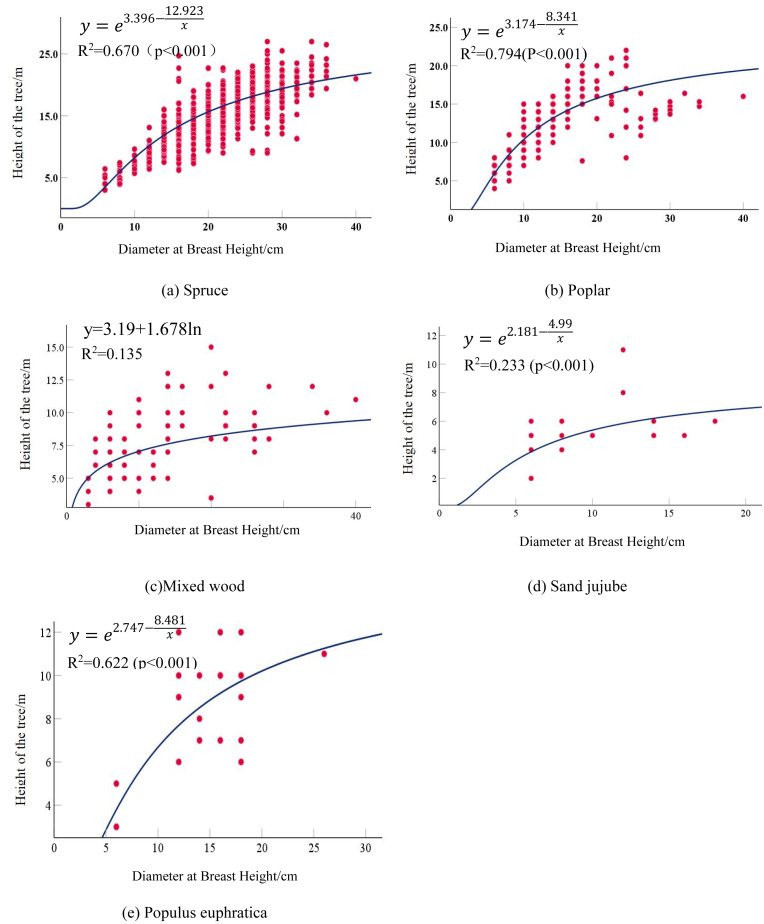
Scatter plot and fitted curve of diameter at breast height-tree height.

Both the regression models for Spruce and Poplar reached a goodness-of-fit also being achieved the best performance. Spruce exhibited the highest correlation coefficient of 0.670 (*p* < 0.01), following an S-curve model, while Poplar had an optimal fitting coefficient of 0.794, with the best-fit model being the S-model. A Mixed wood composed of *Ulmus*, *Salix*, *Fraxinus*, and *Robinia* had a maximum correlation coefficient of 0.138, with the optimal model being the logarithmic model. The correlation between the average DBH and average tree height in the Mixed wood was not strong, attributed to the diversity of tree types. Due to the extremely small sample size, Sand jujube had a maximum correlation coefficient of only 0.208. *Populus euphratica* maintained a stable significance level, with the highest correlation coefficient reaching 0.622, and the optimal model was the S-curve model. The optimal fitting equations for each tree types are as follows in Table 6: the detailed calculation results are presented in Table 7. The optimal fitted curve is shown in Fig. 2.

**Table 6:** Estimation results of parameters for the basic stand age-diameter at breast height model.

Tree type	Optimal fitting equations
Spruce	$y={\mathrm{e}}^{3.396- \frac{12.923}{x} }$
Poplar	$y={\mathrm{e}}^{3.174- \frac{8.341}{x} }$
Mixed wood	*y* = 3.19 + 1.678ln*x*
Sand jujube	$y={\mathrm{e}}^{2.181- \frac{4.99}{x} }$
*Populus euphratica*	$y={\mathrm{e}}^{2.747- \frac{8.481}{x} }$

**Table 7:** Evaluation of the basic model’s accuracy.

Tree species	Equation	*R* ^2^	*F*-value	df_1_	df_2_	*P*-value	Constant	b_1_	b_2_	b_3_
Spruce	Linear	0.519	2,362.782	1	2,191	0.000	4.978	0.519		
	Logarithm	0.569	2,890.426	1	2,191	0.000	−13.977	9.922		
	Quadratic polynomial	0.564	1,420.736	2	2,190	0.000	−2.568	1.305	−0.019	
	Cubic polynomial	0.574	938.954	3	2,189	0.000	−9.467	2.497	−0.082	0.001
	S	0.670	4,455.299	1	2,191	0.000	3.396	−12.923		
	Growth	0.502	2,214.431	1	2,191	0.000	1.876	0.041		
	Reciprocal	0.536	2,527.391	1	2,191	0.000	23.648	−149.972		
	Logistic	0.561	29,300	2	2,190	0.000	0.000	19.582	0.181	11.616
Poplar	Linear	0.546	506.257	1	421	0.000	5.401	0.500		
	Logarithm	0.702	995.966	1	421	0.000	−7.342	7.744		
	Quadratic polynomial	0.779	744.284	2	420	0.000	−2.777	1.658	−0.350	
	Cubic polynomial	0.785	513.53	3	419	0.000	−5.332	2.215	−0.690	0.001
	S	0.794	1,625.69	1	421	0.000	3.174	−8.341		
	Growth	0.538	492.786	1	421	0.000	1.821	0.046		
	Reciprocal	0.733	1,157.464	1	421	0.000	19.773	−86.732		
	Logistic	0.782	489.094	1	421	0.000	0.000	16.631	0.276	8.296
Mixed wood	Linear	0.133	15.376	1	93	0.000	5.539	0.126		
	Logarithm	0.138	16.052	1	93	0.000	3.19	1.678		
	Quadratic polynomial	0.123	7.612	2	92	0.915	5.463	0.138	0.000	
	Cubic polynomial	0.137	5.985	3	91	0.120	3.409	0.630	−0.030	0.000
	S	0.098	11.243	1	93	0.000	2.107	−1.838		
	Growth	0.094	10.780	1	93	0.387	1.697	0.016		
	Reciprocal	0.126	14.529	1	93	0.000	8.817	−14.35		
	Logistic	0.112	11.506	2	92	0.000	0.000	21.493	0.026	40.162
Sand jujube	Linear	0.102	3.044	1	17	0.099	3.371	0.197		
	Logarithm	0.147	4.111	1	17	0.903	0.311	2.252		
	Quadratic polynomial	0.208	3.363	2	16	0.445	−2.815	1.481	−0.058	
	Cubic polynomial	0.164	2.181	3	15	0.688	−7.752	2.970	−0.196	0.004
	S	0.233	6.468	1	17	0.021	2.181	−4.99		
	Growth	0.142	3.984	1	17	0.364	1.164	0.044		
	Reciprocal	0.183	5.072	1	17	0.039	7.914	−22.447		
	Logistic	0.173	3.703	1	17	0.071	0.000	6.308	0.602	5.135
*Populus euphratica*	Linear	0.345	12.069	1	20	0.002	3.419	0.348		
	Logarithm	0.441	17.559	1	20	0.201	−3.960	4.758		
	Quadratic polynomial	0.415	8.459	2	19	0.714	−1.049	1.040	−0.024	
	Cubic polynomial	0.478	7.401	3	18	0.087	−12.233	3.855	−0.226	0.004
	S	0.622	35.559	1	20	0.000	2.747	−8.481		
	Growth	0.409	15.505	1	20	0.305	1.266	0.056		
	Reciprocal	0.493	21.407	1	20	0.000	12.543	−51.315		
	Logistic	0.484	15.049	2	19	0.000	0.000	9.294	0.664	6.481

#### Simulation results of the basic model for tree age-diameter at breast height (DBH)

**Figure 3:**
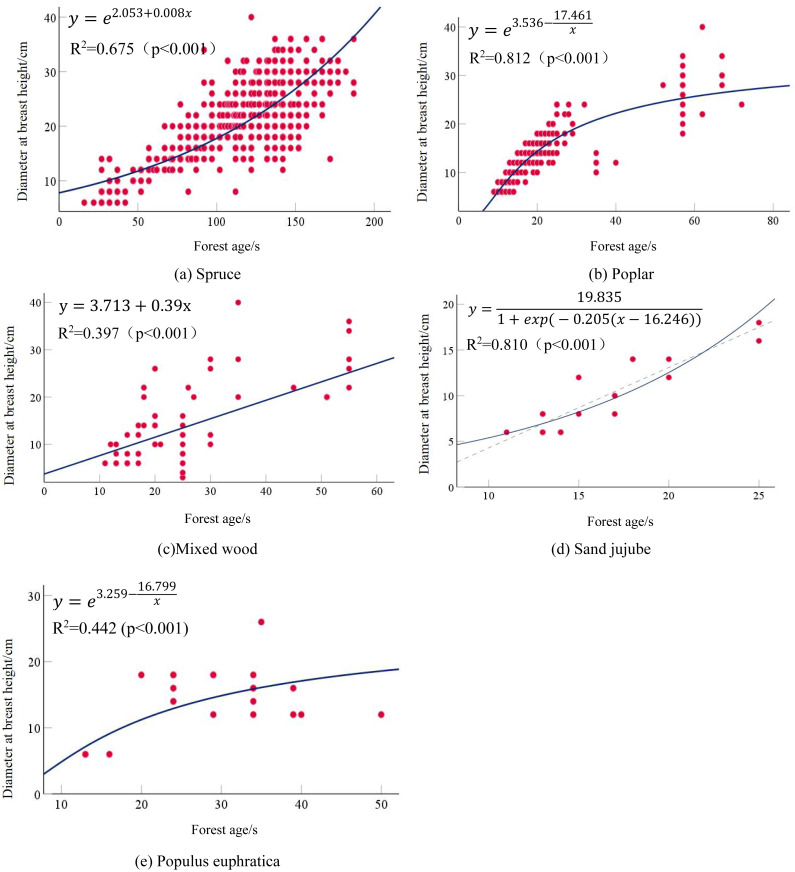
Age-diameter scatter plot and fitted curves.

All stand age-DBH models reached a significant level (*P* < 0.001). The growth model yielded the highest coefficient of determination (*R*^2^ = 0.675) and thus best performed among all models tested. For Poplar, the stand age-average DBH models also reached the best-fit model being the S-curve model, which had an *R*^2^ = 0.812 (*P* < 0.001). This indicated that as forest age increased, the average DBH of Poplar also increased, demonstrating a good correlation between the two. The growth models fitted for mixed wood all reached the highest coefficient of determination reaching *R*^2^ = 0.397 (*P* < 0.001), and the optimal growth model belonged to the linear model. Overall, due to the heterogeneity of tree types in Mixed wood and the relatively small number of modeling samples, the coefficients of determination were not high overall. The forest age-average DBH growth models for Sand jujube all with good overall fit and the highest model coefficient reaching *R*^2^ = 0.810. The optimal growth model belonged to the Logistic model. The stand age-average DBH growth models for *Populus euphratica* reached a significant level with *P* < 0.005. Compared with other tree types, the fitting coefficients for *Populus euphratica* were lower, with the highest coefficient of determination being *R*^2^ = 0.442, and the optimal model was the S-model. This may be attributed to the smaller number of modeling samples for *Populus euphratica*. The optimal models for the relationship between stand age and average DBH for each tree types are Respectively,as shown in Table 8: the detailed calculation results are presented in Table 9. The optimal fitted curve is shown in Fig. 3.

**Table 8:** Statistical analysis of sample plot factors and climatic factors.

Tree type	Optimal fitting equations
Spruce	*y* = e^2.053+0.008*x*^
Poplar	$y={\mathrm{e}}^{3.536- \frac{17.461}{x} }$
Mixed wood	*y* = 3.713 + 0.39*x*
Sand jujube	$y= \frac{19.835}{1+\exp (-0.205(x-16.246))} $
*Populus euphratica*	$y={\mathrm{e}}^{3.259- \frac{16.799}{x} }$

**Table 9:** The correlation between diameter at breast height and random variables.

Tree species	Equation	*R* ^2^	*F*-value	df_1_	df_2_	*P*-value	Constant	b_1_	b_2_	b_3_
Spruce	Linear	0.621	3,582.459	1	2,191	0.000	4.232	0.144		
	Logarithm	0.592	3,183.417	1	2,191	0.000	−37.027	12.291		
	Quadratic polynomial	0.624	1,818.380	2	2,190	0.000	1.741	0.000	0.198	
	Cubic polynomial	0.626	1,221.956	3	2,189	0.000	4.819	−4.430E−6	0.001	0.078
	S	0.629	3,712.620	1	2,191	0.000	3.434	−45.364		
	Growth	0.675	4,547.180	1	2,191	0.000	2.053	0.008		
	Reciprocal	0.456	1,838.151	1	2,191	0.000	27.527	−705.446		
	Logistic	0.646	3,993.550	1	2,191	0.000	0.000	30.816	0.022	79.050
Poplar	Linear	0.692	949.978	1	421	0.000	5.254	0.396		
	Logarithm	0.782	1,511.094	1	421	0.000	−20.406	11.597		
	Quadratic polynomial	0.760	669.191	2	420	0.000	−1.582	0.940	−0.008	
	Cubic polynomial	0.785	515.747	3	419	0.000	−8.583	1.829	−0.040	0.000
	S	0.812	1,820.469	1	421	0.000	3.536	−17.461		
	Growth	0.548	512.187	1	421	0.000	1.980	0.026		
	Reciprocal	0.737	1,183.852	1	421	0.000	26.735	−227.127		
	Logistic	0.752	150	1	421	0.000	0.000	25.470	0.120	17.830
Mixed wood	Linear	0.397	62.994	1	93	0.000	3.713	0.390		
	Logarithm	0.389	60.898	1	93	0.000	−21.727	11.304		
	Quadratic polynomial	0.391	31.204	2	92	0.000	2.731	0.463	−0.001	
	Cubic polynomial	0.388	20.826	3	91	0.000	−4.299	1.312	−0.032	0.000
	S	0.278	37.225	1	93	0.000	3.287	−17.866		
	Growth	0.322	45.559	1	93	0.000	1.742	0.027		
	Reciprocal	0.348	51.102	1	93	0.000	26.093	−259.393		
	Logistic	0.351	51.894	1	93	0.000	0.000	13.968	−2.778e+06	2.675e+07
Sand jujube	Linear	0.807	76.442	1	17	0.000	−4.521	0.881		
	Logarithm	0.804	74.610	1	17	0.000	−32.374	15.260		
	Quadratic polynomial	0.798	36.660	2	16	0.000	−8.276	1.318	−0.012	
	Cubic polynomial	0.799	36.806	3	15	0.000	−7.334	1.129	0.000	0.000
	S	0.768	60.554	1	17	0.000	3.783	−24.326		
	Growth	0.757	57.093	1	17	0.000	0.840	0.084		
	Reciprocal	0.771	61.438	1	17	0.000	25.718	−246.566		
	Logistic	0.810	62.751	1	17	0.000	0	19.835	0.205	16.246
*Populus euphratica*	Linear	0.112	2.592	1	20	0.123	9.452	0.171		
	Logarithm	0.174	5.438	1	20	0.030	−5.862	6.122		
	Quadratic polynomial	0.417	8.495	2	19	0.002	−10.525	1.675	−0.026	
	Cubic polynomial	0.415	6.855	3	18	0.003	−32.355	4.235	−0.115	0.001
	S	0.442	17.640	1	20	0.000	3.259	−16.799		
	Growth	0.157	4.918	1	20	0.04	2.074	0.018		
	Reciprocal	0.280	9.173	1	20	0.007	20.969	−168.609		
	Logistic	0.422	69.800	1	20	0.000	0.000	15.846	0.443	14.931

#### Evaluation of the basic model’s accuracy

The optimal DBH—tree height models and stand age-DBH models for each dominant tree types in the Manas River Basin of Xinjiang were calculated. The precision of the models fitted to the validation data is shown in Table 10 below: for the single-types models, *Populus euphratica* exhibited the smallest values across all three indicators, indicating a relatively higher prediction accuracy and the best fitting effect. The Mixed wood models showed larger values for mean error, mean relative error, and root mean square error, indicating a relatively lower prediction accuracy and the worst fitting effect. Considering both the DBH-tree height and stand age-DBH models, although Sand jujube had smaller mean error and mean relative error, *Populus euphratica* maintained a low level of root mean square error and, compared to other types models, demonstrated more stable overall error performance. Therefore, comprehensive analysis suggests that *Populus euphratica* had the best prediction accuracy among these types. This may be due to its growth characteristics or environmental factors that make its growth data more consistent with the prediction models. The Mixed wood had the worst prediction accuracy, which is related to the diversity of tree types it contains and the heterogeneity of the data.

**Table 10:** Multivariate nonlinear forest growth model.

Fitted model	Tree species	Mean error	Mean relative error	Root mean square error
Height-DBH model	Spruce	12.340	0.847	12.819
	Poplar	1.357	0.121	1.893
	Mixed wood	7.774	1.518	8.367
	Sand jujube	1.879	0.255	3.036
	*Populus euphratica*	0.978	0.139	1.198
Age-DBH model	Spruce	2.490	2.490	3.330
	Poplar	1.560	1.560	2.110
	Mixed wood	8.290	8.290	11.860
	Sand jujube	0.950	0.950	1.100
	*Populus euphratica*	1.280	0.087	1.590

### Selection of parameters and results for multivariate nonlinear forest growth models

#### Parameter selection for multivariate nonlinear forest growth models

By calculating the correlation coefficient R between DBH and various random factors, the specific results are shown in Table 11: the correlation coefficient R between elevation data (Km) and DBH is 0.520, which is the largest absolute value of positive correlation coefficients among the Environmental Covariates, indicating the strongest correlation between elevation data and DBH. Next, annual precipitation (P) and minimum of maximum daily temperature (TXn) have relatively strong correlations with tree DBH, with correlation coefficients R both being 0.410. The correlation coefficient R between vapor pressure deficit (KPa) and DBH is—0.060, which is the smallest absolute value of the correlation coefficients listed, suggesting the weakest correlation between vapor pressure deficit and DBH. Based on the comparison of correlation coefficients, the three Environmental Covariates with the strongest correlations with DBH—elevation data (DEM), annual precipitation (P), and minimum of maximum daily temperature (TXn)—are introduced into the basic model for the construction of a multivariate nonlinear forest growth model. Prior to constructing the multivariate nonlinear growth models, we performed multicollinearity diagnostics for all candidate independent variables. The variance inflation factor (VIF) method was used to assess the degree of collinearity among the variables. The results indicated that the VIF values for all variables were below the threshold of 5 (specific values: DEM = 2.3, Annual Precipitation = 2.134, TXn = 1.676), demonstrating the absence of severe multicollinearity issues. Therefore, these variables were deemed suitable for simultaneous inclusion in the model for analysis.

**Table 11:** Multivariate nonlinear forest growth model.

Variable	Variable symbol	*R*
Diameter at breast height/km	DEM	0.520
Annual precipitation/mm	P	0.410
Vapor pressure deficit/kpa	VPD	−0.060
Maximum temperature extreme/°C	TXx	−0.420
Lowest maximum temperatur/°C	TXn	0.410
Lowest minimum temperature/°C	TNn	0.350
Highest minimum temperature/°C	TNx	−0.360
Mean temperature difference/°C	DTR	−0.167
Peak daily temperatures in summer/°C	SU	−0.460
Daily highs during icy days/°C	ID	0.400
Duration of warm days/day	WSDI	0.060
Duration of continuous cold days/day	CSDI	−0.340

#### Simulation results of the multivariate nonlinear forest growth model

Based on the basic model, the three environmental covariates with the strongest correlations with DBH—elevation data (DEM), annual precipitation (P), and minimum of maximum daily temperature (TXn)—are introduced to construct a multivariate nonlinear forest growth model. Ultimately, the multivariate nonlinear forest growth models for each dominant tree types are determined. The specific results are shown in Table 12 below:

**Table 12:** Evaluation of the accuracy of multivariate nonlinear forest growth models.

Model name	Dominant tree species	Multivariate non1inear forest growth model
DBH-Tree Height Model	Spruce	$y={\mathrm{e}}^{3.99- \frac{12.877}{{x}_{1}} -0.0009{x}_{2}-0.27{x}_{3}-0.12{x}_{4}}$
	Poplar	$y={\mathrm{e}}^{3.174- \frac{8.341}{{x}_{1}} -0.003{x}_{2}+0.18{x}_{3}+0.01{x}_{4}}$
	Mixed wood	*y* = 3.19 + 1.678ln*x*_1_ − 0.05*x*_2_ + 0.27*x*_3_ + 0.03*x*_4_
	Sand jujube	$y={\mathrm{e}}^{2.181- \frac{4.99}{{x}_{1}} +0.006{x}_{2}-0.14{x}_{3}-0.03{x}_{4}}$
	*Populus euphratica*	$y={\mathrm{e}}^{2.747- \frac{8.481}{{x}_{1}} -0.003{x}_{2}+1.89{x}_{3}-0.08{x}_{4}}$
Age-DBH Model	Spruce	*y* = *e*^2.053+0.008*x*_1_+0.0008*x*_2_+0.62*x*_3_−0.03*x*_4_^
	Poplar	$y={e}^{3.536- \frac{17.461}{{x}_{1}} -0.003{x}_{2}+0.12{x}_{3}+0.03{x}_{4}}$
	Mixed wood	*y* = 3.713 + 0.39x_1_+0.003x_2_-0.86x_3_-0.12x_4_
	Sand jujube	$y= \frac{19.835}{1+\exp (-0.205({x}_{1}-16.246))-0.004{x}_{2}+0.79{x}_{3}+0.13{x}_{4}} $
	*Populus euphratica*	$y={e}^{3.259- \frac{16.799}{{x}_{1}} +0.056{x}_{2}-1.25{x}_{3}-0.3{x}_{4}}$

The calculation results indicate that the inclusion of Environmental Covariates has improved the model accuracy for dominant tree types overall, but a decreasing trend in model accuracy is observed for individual types. The specific results are shown in Table 13: The root mean square errors (RMSE) for DBH-tree height have decreased to some extent. For example, the RMSE for Spruce has decreased from 12.819 to 2.390, that for Poplar has decreased to 1.710, the RMSE for Mixed wood has reduced from 8.367 to 2.010, the RMSE for Sand jujube has decreased from 3.036 to 1.740, while the RMSE for *Populus euphratica* has increased by 0.572. For the age-DBH models of dominant tree types, the accuracy of the age-DBH model for Spruce and Mixed wood has improved, decreasing from 3.330 to 3.100 and from 11.860 to 5.410, respectively. However, for Poplar, Sand jujube, and *Populus euphratica*, the RMSEs have increased slightly. In terms of the model evaluation metric R^2^, the R^2^ for the DBH-tree height model of Poplar has increased to 0.800, and the R^2^ for the DBH-tree height of Mixed wood has improved to 0.473. Additionally, the accuracy of the age-DBH model for Mixed wood has increased from 0.397 to 0.571, and the accuracy of the age-DBH model for Sand jujube has risen from 0.810 to 0.890. Overall, the incorporation of random effects has improved the accuracy of the models, but there are certain limitations.

**Table 13:** Calculating the correlation coefficient of a basic model using deep learning methods.

Fitted model	Dominant tree species	*R* ^2^	Mean relative error
DBH-Tree Height Model	Spruce	0.601	2.39
	Poplar	0.800	1.71
	Mixed wood	0.473	2.01
	Sand jujube	0.198	1.74
	*Populus euphratica*	0.561	1.77
Age-DBH Model	Spruce	0.653	3.10
	Poplar	0.784	2.62
	Mixed wood	0.571	5.41
	Sand jujube	0.890	1.28
	*Populus euphratica*	0.269	4.02

#### Evaluation of the accuracy of multivariate nonlinear models

The calculations were performed on a 30% validation sample to obtain the accuracy test values for the multivariate nonlinear DBH-tree height models and multivariate nonlinear age-DBH models for each dominant tree types. The specific results are shown in Table 14 below. In the DBH-tree height models, Mixed wood exhibited larger values for the accuracy assessment coefficients, indicating poorer fitting accuracy. For Sand jujube, the mean error, mean relative error, and root mean square error were all relatively small, indicating the best fitting accuracy. Among the age-DBH model group, Sand jujube had the smallest mean error of 3.420 and the lowest mean relative error of 0.230, demonstrating the best fitting accuracy. Mixed wood had the lowest RMSE of 0.110, indicating good fitting accuracy as well. Overall, Sand jujube showed the best fitting accuracy, while Mixed wood had an average fitting performance.

**Table 14:** Calculating the correlation coefficient of a multivariate nonlinear forest growth model using deep learning methods.

Fitted model	Dominant tree species	Mean error	Mean relative error	Root mean square error
DBH-Tree Height Model	Spruce	16.230	1.000	15.770
	Poplar	11.980	0.980	11.440
	Mixed wood	58.340	9.250	46.440
	Sand jujube	3.640	0.480	−1.400
	*Populus euphratica*	8.150	1.000	8.000
Age-DBH Model	Spruce	21.050	1.000	20.380
	Poplar	20.950	1.530	−16.930
	Mixed wood	5.160	0.460	0.110
	Sand jujube	3.420	0.230	0.190
	*Populus euphratica*	67.300	3.870	−56.950

### Simulation results offorest growth models based on deep learning

In this paper, deep learning methods were used to calculate the correlation coefficients for basic models such as DBH-tree height, age-DBH, and multivariate nonlinear forest growth models. Comparative results showed that deep learning methods exhibited high accuracy in simulating forest growth models. The results of using deep learning to calculate the correlation coefficients for the basic models are shown in Table 15 below: The highest correlation coefficient for the basic DBH-tree height model reached 0.810, and the highest correlation coefficient for the basic age-DBH model reached 0.860. From the perspective of tree types, deep learning provided the best fitting accuracy for Poplar in the basic models. The results of using deep learning to calculate the correlation coefficients for multivariatenonlinear forest growth models are shown in Table 16: The correlation coefficient for Poplar in the age-DBH model was the highest, reaching 0.920, indicating that the model has a very strong predictive ability for the relationship between Poplar’s age and DBH. The lowest correlation coefficient was 0.670, which appeared for Sand jujube in the DBH-tree height model, suggesting that this method has a relatively weaker predictive ability for the relationship between *Sand jujube*’s DBH and tree height, and improvements may be needed in model optimization. From the perspective of tree types, in the fitting of multivariate nonlinear models using deep learning, Poplar showed the best fitting effect, while sand jujube had a poor fitting effect. In summary, deep learning methods exhibit higher accuracy in forest growth models.

**Table 15:** Calculating the correlation coefficient of a basic model using deep learning methods.

Basic model	Dominant tree species	*R* ^2^
DBH-Tree Height Model	Spruce	0.590
	Poplar	0.810
	Mixed wood	0.410
	Sand jujube	0.610
	*Populus euphratica*	0.550
Age-DBH Model	Spruce	0.710
	Poplar	0.860
	Mixed wood	0.560
	Sand jujube	0.160
	*Populus euphratica*	0.030

**Table 16:** Calculating the correlation coefficient of a multivariate nonlinear forest growth model using deep learning methods.

Multivariate nonlinear forest growth model	Dominant tree species	*R*_2_
DBH-Tree Height Model	Spruce	0.690
	Poplar	0.860
	Mixed wood	0.690
	Sand jujube	0.670
	*Populus euphratica*	0.790
Age-DBH Model	Spruce	0.720
	Poplar	0.920
	Mixed wood	0.760
	Sand jujube	0.960
	*Populus euphratica*	0.760

Although this study did not conduct formal quantification of variable importance, we can make preliminary inferences about the roles of key predictors through correlation analysis and ecological principles. For Poplar trees as riparian species, their characteristics suggest that moisture conditions may be the critical limiting factor, consistent with the observed positive correlation between annual precipitation and growth (*r* = 0.41). In contrast, as mountainous species, Spruce growth appears more constrained by temperature conditions, as evidenced by the strong correlation between altitude and growth (*r* = 0.52). While these patterns require further verification, they provide preliminary clues to understanding the differential growth drivers of types in this region.

## Discussion

### Comparison of basic model simulation results

Based on forest inventory data from the Manas River basin in Xinjiang, this article constructs basic growth models for different dominant tree types, including diameter-at-breast-height (DBH)-tree height and age-DBH. The results indicate that there is no correlation between the accuracy of growth models and the optimal models for different dominant tree types. The fitting accuracy and optimal basic models vary among different tree types in the study area. Among them, *Populus euphratica* exhibits the best fitting effect, primarily because it has adapted to the arid climate, salt-tolerant soil, and relies on natural conditions such as groundwater and seasonal floods to survive and reproduce in the Manas River basin of Xinjiang, demonstrating strong adaptability to external disturbances. Secondly, the basic model fitting error for Poplar is also relatively low, indicating good performance and strong predictive ability. The fitting effect of Sand jujube is also good, which is mainly related to its drought and heat tolerance and its non-strict requirements for soil type. Even for the same tree types in the same study area, different growth states may occur due to factors such as precipitation, temperature, soil properties, and human disturbances. Therefore, the fitting accuracy of the models mainly depends on the model type and tree types differences. Similar conclusions have been drawn in other studies. For example, based on research conducted in Maoershan, the optimal model for the relationship between height and DBH (diameter at breast height) of Spruce trees is the power function model, with a coefficient of determination exceeding 0.980 (Han, Zhou & Qi, 2019). Taking Guangxi Zhuang Autonomous Region as the study area, it was found that the Chapman-Richards model is the best basic model for the relationship between tree height and DBH (diameter at breast height) of Chinese fir (Lu et al., 2015). Stand dominant height, stand basal area, and annual average precipitation are significantly correlated with tree height growth. The coefficient of determination for the fitted model of Spruce tree height and DBH (diameter at breast height) based on Changbai Mountain is 0.787 (Liu, Mao & Li, 2016). Studies have shown that the differences in growth status among different tree types are the result of a combination of factors such as their genetic characteristics, environmental adaptability, human intervention, and natural disasters (Wang, Zhang & Zhen, 2020). In the Manas River basin, the differences in growth status among *Populus euphratica*, Sand jujube, and Poplar are mainly due to their varying abilities to adapt to drought, salinity, water availability, and soil conditions.

### Comparison of simulation results for multivariate nonlinear forest growth models

**Figure 4:**
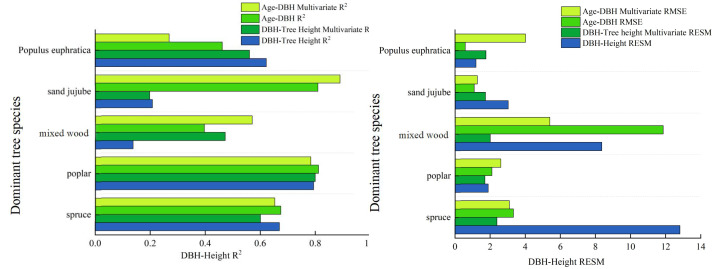
Comparison chart of accuracy between the basic model and the multivariate nonlinear model.

By introducing three Environmental Covariates: altitude, annual precipitation, and minimum of daily maximum temperature, which have the strongest correlation with DBH (diameter at breast height)—we established multivariate nonlinear growth models for different dominant tree types in the study area. The primary objective of this study is to evaluate the predictive performance of different modeling approaches—such as basic models, multivariate nonlinear forest growth models, and deep learning methods—in simulating forest growth relationships. The inclusion of climate and topographic factors aims toenhance model interpretability and prediction accuracy, rather than to assert their inherent physiological mechanisms. The results show that there is a significant positive correlation between DBH and altitude, annual precipitation, and minimum of daily maximum temperature for dominant tree types in the Manas River basin. In contrast, there is a significant negative correlation between DBH growth and maximum temperature, as well as daily maximum temperature in summer. Climate change profoundly affects tree growth (Huang, Yves & Frank, 2013; Bai, Chang & Zhang, 2016), the introduction of has improved the accuracy of some models but also reduced the accuracy of others. The introduction of climate and topographic variables revealed species-specific variations in the accuracy of forest growth models. For types like Spruce and Mixed wood, the model precision R^2^ showed significant improvement, indicating that environmental factors play a crucial role in explaining their growth dynamics. However, for species such as Poplar, model accuracy declined (*e.g.*, the R^2^ of the Poplar DBH-height model dropped from 0.622 to 0.561). This discrepancy may stem from different environmental response mechanisms across species: As an extremely drought-tolerant species, Poplar’s growth likely depends more on local critical factors like deep groundwater—key elements not included in this model—and demonstrates lower sensitivity to conventional climatic indicators such as annual precipitation. Therefore, when developing climate-sensitive growth models, it is essential to account for ecological trait differences among tree types. This is similar to the observation made by Zhang (2015) that an increase in maximum precipitation during the growing season can inhibit the growth of Korean pine. The main reason may be that excessive rainfall leads to high humidity in the air and reduced solar radiation. Additionally, excessive rainfall intensifies the leaching of soil nutrients, which is detrimental to the accumulation of nutrients in trees. Additionally, The model results showed a positive correlation between tree breast height diameter and TXn. This statistical association can be explained ecologically: studies have shown that the extra heat generated by moderate warming may contribute to tree growth under certain conditions, and warmer climatic conditions may also extend the growing season of trees, (Ma, 2024a; Ma, 2024b) which is beneficial to their growth. This also validates that the inclusion of environmental covariates in this study improves the accuracy of the models. Rapid warming can slow the growth of *Larix gmelinii* on hillsides, while in valleys, due to the melting of permafrost, the growth of *Larix gmelinii* accelerates in the short term (Liang, Sun & Li, 2021), this is similar to the results of this study, where DEM (Digital Elevation Model) significantly influences tree growth processes. Tree types growing at different altitudes have different requirements for growth conditions. Trees at different latitudes are also affected in their growth: Radial growth of trees in high-latitude regions increases significantly, while radial growth in mid- and low-latitude regions is not pronounced (Ou & Quiñónez Barraza, 2023). Overall, individual tree growth models that incorporate climate factors and tree size diversity factors can accurately predict the dynamic changes in individual tree growth (Roy & Debbarma, 2024), however, since tree growth is a complex and comprehensive process, further in-depth research is needed. In summary, the multivariate nonlinear forest growth model exhibits more stable accuracy compared to the basic model, as illustrated specifically in Fig. 4: Analyzing the diameter-at-breast-height (DBH)-tree height model, the multivariate nonlinear forest growth model demonstrates a significant increase in accuracy (R^2^), a reduction in the root mean square error (RMSE), and an improved fit compared to the basic model. From the analysis of the stand age *versus* DBH model, the multivariate nonlinear forest growth model showcases a higher R^2^ and a lower RMSE.

### Comparison of simulation results using deep learning methods

**Figure 5:**
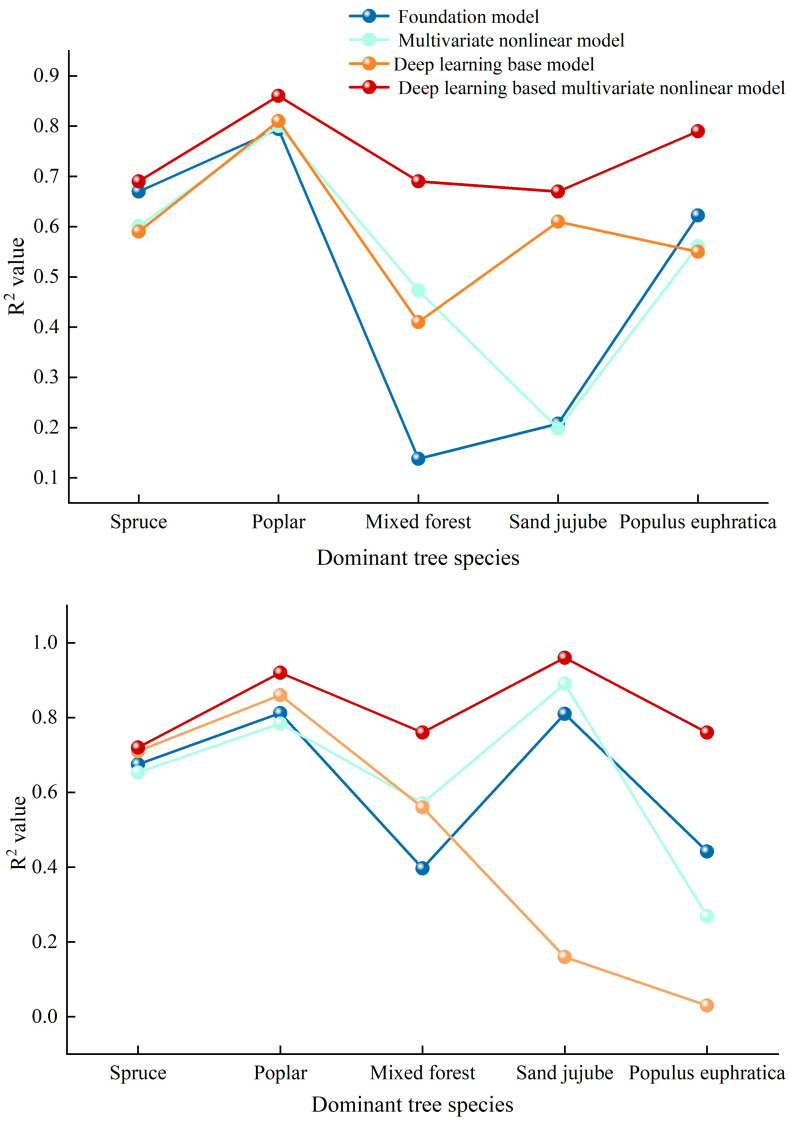
Comparative diagrams of DBH-height and stand age-DBH growth models. (A) Comparison chart of correlation coefficients among various diameter at breast height (DBH) and tree height models (B) Comparison Chart of Correlation Coefficients Among Various Stand Age-Diameter at Breast Height (DBH) Models.

This article employs deep learning to calculate the correlations between DBH and tree height, as well as stand age and DBH, in the Manas River Basin. The results indicate that the fitting accuracy achieved using deep learning algorithms is significantly higher than that of the basic models, as specifically shown in Fig. 5A: The correlation coefficients of the multivariate nonlinear forest growth models for DBH—tree height, calculated using deep learning methods, are generally higher than those of the basic models, suggesting that the deep learning approach yields the highest prediction accuracy. Among these, the R^2^ values of the deep learning models for Spruce, Poplar, Sand jujube, and *Populus euphratica* are significantly higher than those of the basic models. For Mixed wood, the R^2^ value of the deep learning model also increases, albeit with a relatively smaller gap. Examining the stand age-DBH model diagram (b) calculated using deep learning methods, the accuracy of the deep learning-optimized multivariate nonlinear model fluctuates between 0.700 and 0.900, while the optimized basic model fluctuates between 0.030 and 0.860. Overall, the accuracy of the deep learning-optimized model is higher than that of both the basic model and the unoptimized multivariate nonlinear model. The application of deep learning methods in forest growth models demonstrates significant advantages, especially in handling nonlinear relationships, improving prediction accuracy, and adapting to complex data. Compared to basic models, 500 deep learning models are better able to capture the complexity of forest growth, providing more reliable scientific data for forest resource management, ecological restoration, and climate change research. This is consistent with findings from a DLA (Deep Learning Algorithm) model for Chinese fir tree height-DBH (diameter at breast height) constructed through deep learning algorithms, which revealed that the model constructed using deep learning algorithms had slightly higher fitting accuracy than traditional generalized tree height-DBH models (Liu et al., 2024). Ou & Quiñónez Barraza (2023) used neural network methods to establish a tree height-DBH model for Durango pine based on data from 1,000 survey plots of Mixed wood in Mexico, which also indicated that the growth model established through deep learning had higher accuracy.

### Limitations and outlook

Based on the construction of growth models for dominant tree types in the Manas River Basin of Xinjiang using various basic models, this article introduces three climatic factors to establish multivariate nonlinear growth models for these tree types, thereby improving the accuracy of the basic models. However, due to the difficulty in obtaining data, only the 2011 continuous forest inventory data were used in this article. Deep learning was only applied to calculate the fitting accuracy of the basic model and the random effects model, without conducting predictions or more in-depth research. Meanwhile, giving that the data originates from a single-year forest resource inventory, this study has limitations in revealing the long-term dynamic impacts of climatic factors on tree growth. The relationships observed in the model primarily reflect static pattern correlations, and their causal mechanisms require further exploration through long-term observational studies or process-based growth models such as 3-PG and BIOME-BGC in future research. In future work, we will explore the application of deep learning models in predicting tree growth (Dutta Roy & Debbarma, 2024; Liu et al., 2024), gaining a deeper understanding of the dynamic processes of forest ecosystems, and providing important scientific tools for forest resource management, ecological restoration, climate change adaptation, and economic decision-making.

## Conclusion

This study systematically constructed and evaluated basic, multivariate nonlinear, and deep learning-optimized forest growth models for five dominant tree types in the arid Manas River Basin of Xinjiang. The primary objective was to identify the most effective modeling approach for characterizing DBH-height and age-DBH relationships under complex environmental influences.

(1) The key findings demonstrate that no single model type universally outperforms others across all tree types. Instead, the optimal model is highly dependent on the biological characteristics and environmental adaptability of each tree types. For instance, *Populus euphratica* exhibited the highest accuracy with basic models, likely due to its stable growth pattern in arid conditions. In contrast, the growth of Poplar was best captured by deep learning models, highlighting its sensitivity to environmental factors that nonlinear methods can elucidate. The integration of climatic and topographic variables (DEM, precipitation, TXn) generally enhanced model performance, validating the importance of including external drivers in growth predictions for this region. However, the varied responses across species underscore the complexity of forest ecosystems and the necessity of species-specific modeling strategies.

(2) Beyond methodological comparisons, our results carry significant practical implications for forest management. The high-accuracy models developed here can be directly integrated into decision-support systems for the Manas River Basin. For example, forest managers could use the deep learning models for Poplar to predict timber yield under different climate scenarios or utilize the robust basic models for *Populus euphratica* to monitor the health of this ecologically critical species. Furthermore, the identified strong correlations between DBH and elevation/precipitation provide actionable insights for designing targeted conservation strategies, such as prioritizing reforestation in areas with optimal growth conditions.

(3) Despite these contributions, this study has limitations, primarily the reliance on a single year’s (2011) forest inventory data, which restricts the analysis of temporal dynamics. Future work should focus on incorporating multi-temporal datasets to develop predictive growth models under climate change scenarios. Additionally, applying the deep learning framework to forecast long-term growth trends and integrating these models with remote sensing data for large-scale application present promising research directions. In conclusion, this research provides a versatile modeling framework and a solid scientific basis for achieving sustainable forest management and enhancing ecological security in arid regions like the Manas River Basin.

##  Supplemental Information

10.7717/peerj.20650/supp-1 Supplemental Information 1 Construction of multivariate nonlinear forest growth models This code constructs a multivariate nonlinear forest growth model, though the current segment demonstrates only a multivariate linear model example. The core code implemented in this work employs deep learning methodologies to compute model accuracy metrics, thereby investigating the application of deep learning techniques in forest growth modeling.

10.7717/peerj.20650/supp-2 Supplemental Information 2 In-depth academic mould preferential education This code segment primarily constructs a neural network model to optimize the forest growth model, with results indicating satisfactory model fitting performance

10.7717/peerj.20650/supp-3 Supplemental Information 3 Survey data on forest resources The 2011 Xinjiang Forest Resource Inventory data used in this study for the Manas River Basin in Xinjiang, including basic information such as forest type, forest age, and tree height, along with additional factors (e.g., precipitation) incorporated subsequently to assess their influence on forest growth.
This dataset contains fundamental forest characteristics including stand age, tree height, and diameter at breast height (DBH), playing a crucial role in sustainable forest management studies.

## Additional Information and Declarations

### Data Availability

The following information was supplied regarding data availability: The code and raw data are available in the Supplemental Files.
